# Quantification of CD4 Recovery in Early-Treated Infants Living With HIV

**DOI:** 10.1097/QAI.0000000000002905

**Published:** 2022-01-07

**Authors:** Juliane Schröter, Anet J. N. Anelone, Rob J. de Boer

**Affiliations:** a Theoretical Biology & Bioinformatics, Utrecht University, Utrecht, the Netherlands; and; b Currently, School of Mathematics and Statistics, University of Sydney, Sydney, Australia.

**Keywords:** perinatal HIV, early antiretroviral therapy, lymphocytes, immune recovery, quantification, mathematical model

## Abstract

**Background:**

Perinatally HIV-acquired infants benefit from an early antiretroviral treatment initiation. Thanks to a short viral exposure time, their immune system can be maintained or reconstituted, allowing a “normal” immune development.

**Methods:**

In this study, we mathematically modeled and quantified individual CD4^+^ T-cell reconstitution of a subset of 276 children who started treatment within 6 months of age and achieved sustained viral suppression. Considering natural age differences in CD4^+^ T-cell dynamics, we fitted distances to age-matched healthy reference values with a linear model approaching an asymptote.

**Results:**

Depleted CD4^+^ percentages (CD4%) and CD4^+^ counts (CD4ct) restored healthy levels during treatment. CD4ct recovered with a median rate of 4 cells/*µL*/d, and individual recovery rates were correlated negatively with their initial CD4ct. CD4 values at onset of treatment decrease with age, whereas recovery times and levels seem to be age-independent. CD4 recovery correlates positively with viral suppression, and the stabilization of CD4 levels usually occurs after viral suppression. CD4 levels stabilize within 3–13 months after treatment initiation. The recovery dynamics of the CD4% is comparable with those of the CD4ct.

**Conclusions:**

In early-treated children with successful viral suppression, the CD4 depletion is typically mild and CD4^+^ T cells tend to “fully” recover in numbers.

## Abstract 2

Supplemental Digital Content is Available in the Text.

## INTRODUCTION

Current WHO guidelines recommend starting antiretroviral treatment (ART) in perinatally HIV-acquired children as early as possible. Although in the past, critical CD4% levels, or a deteriorated health status, were used as criteria to initiate treatment, nowadays the presence of HIV is tested genetically, and a positive HIV diagnosis after birth usually leads to immediate treatment initiation. Early ART initiation not only reduces viral exposure time, and limits the establishment of latent HIV reservoirs, but probably also leads to either maintenance of the immune system or a better capacity for the regeneration of depleted CD4^+^ T cells.^1^ Thus, early treatment initiation in perinatally HIV-acquired infants allows for a fairly normal development of their immune system.

Previous analyses have highlighted that children are better in regenerating their immune system than adults. This is probably because of increased thymic output and an increased CD4^+^ T-cell proliferation, which accelerate CD4^+^ T-cell count (CD4ct) recovery.^2–4^ Thus, the efficiency of T-cell reconstitution after treatment initiation is expected to depend on the age at which treatment is initiated. Furthermore, progressed HIV infections result in low CD4 levels at treatment initiation, which have been associated with poor CD4 reconstitution and reduced maintenance.^4–6^ Very early treatment initiation may therefore lead to rapid and durable immune reconstitution, which could open the opportunity for scheduled treatment interruptions and the possibility for a life without daily treatment for early-treated, perinatally HIV-acquired infants.^1,7–12^

In this study, we aimed to quantify CD4 reconstitution in early-treated, perinatally HIV-acquired infants who achieved successful viral suppression by using mathematical modeling. We correct for the natural CD4 decline in children by normalizing CD4 levels to age-matched reference values from healthy children.^13^ This quantification showed that both depleted CD4ct and CD4% recover to healthy levels and that stable CD4 levels are typically reached after viral suppression. As a consequence, late viral suppression, for example, because of poor adherence to ART, leads to late CD4 recovery.

## MATERIALS AND METHODS

### Data Selection

We studied the reconstitution of CD4 levels (CD4ct and CD4% within total lymphocytes) in infants, from the database of the European Pregnancy and Paediatric Infections Cohort Collaboration (EPPICC), starting standard ART (bPI or NNRTI + 2–3 NRTI) within 6 months of age (N = 469).^14–16^ We only considered children who successfully suppressed the virus [ie, achieved 2 consecutive viral loads (VLs) <400 copies/mL, N = 276] within the follow-up time of EPPICC.^16^ We defined a period of sustained viral suppression as the time having no VL measurements above 400 copies/mL once virally suppressed, and we neglected the fact that some infants achieved viral suppression a second time after a viral rebound, for example, because of treatment complications. We restricted our analyses to those observations obtained during the first period of sustained viral suppression. This period begins at ART initiation, although for some infants we use data from up to 10 days before ART initiation, to enrich baseline measurements. The period ends either at the end of follow-up or at the last measurement before the VL rebounds above 400 copies/mL. For a subset of 58 infants belonging to the Collaborative HIV Paediatric Study (CHIPS) cohort,^17^ we also had data on total lymphocyte counts (TLCs). Their recovery was quantified in a similar fashion.

### CD4 Normalization

TLC and CD4ct in healthy children follow a natural decline.^18,19^ To compare CD4 levels in infants at different ages, we used the normalization functions provided by Schröter et al^13^ as a reference for HIV unexposed (“healthy”) infants. For each CD4^+^ T-cell measurement, we calculated the age-matched healthy median reference CD4 level (CD4_ref_) and calculated the deviation of the measurement from this reference value:

$$\Delta \text{CD}4\;=\text{ CD}4-{\text{CD}4}_{\text{ref}}$$

**FIGURE 1.**
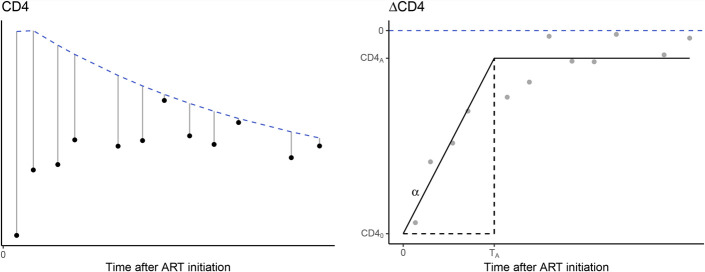
Schematic illustration of CD4 normalization step and the recovery model. In the left panel, black bullets show the original CD4 measurements. In gray, the distances to healthy CD4 reference values (CD4_ref_) are depicted. In the right panel, these distances are presented by the gray bullets (∆CD4). The black dashed lines represent the healthy median CD4 values retrieved from Schröter et al.^13^ The median healthy CD4 values are used for the CD4 transformation and represent the horizontal black dashed line at zero in the right part. The right part shows ∆CD4, on which basis the fitting has been performed. An example model fit is illustrated by the black solid line. The model is fitted according to Equation (1), resulting in CD4_0_, *α*, and T_A_. The asymptote CD4_A_ can be calculated from these 3 parameters. The dashed black lines highlight the CD4 recovery phase that ends at time T_A_, when the asymptote CD4_A_ is reached.

We refer to this difference/distance as the ∆CD4, which is negative if the CD4 measurement is below CD4_ref_, positive if above, and zero if it is matching CD4_ref_ (Fig. 1). We define a ∆CD4 for both the CD4ct and the CD4%. The same normalization procedure is applied to the TLC data.

### Mathematical Model

Visual inspection of the data (see Fig. S1, Supplemental Digital Content, http://links.lww.com/QAI/B786) suggested that ∆CD4 dynamics after ART initiation can be described empirically as a linear approach to a stable asymptote. Therefore, at a given time after ART initiation (t in days), we defined the ∆CD4 by the following recovery model:

(1) $$\begin{array}{l} \Delta \text{CD}4\left(\text{t}\right)\;=\;\Delta {\text{CD}4}_{\text{0}}+\alpha \text{t, if t }\leq {\text{ T}}_{\text{A,}}\\ \Delta \text{CD}4\left(\text{t}\right)\;=\;\Delta {\text{CD}4}_{\text{0}}+\alpha {\text{T}}_{\text{A}}=\Delta {\text{CD}4}_{\text{A}}\text{, if t }>{\text{ T}}_{\text{A,}} \end{array}$$

where ∆CD4_0_ is the difference at the start of treatment, *α* is the recovery rate in either cells per microliter day (for CD4 counts) or in percentages per day (for CD4%), and T_A_ is the time at which ∆CD4 stabilized at the long-term asymptote ∆CD4_A_ (see Fig. 1). We maximized ∆CD4 to the negative reference value CD4_ref_ to prevent negative CD4 levels, that is,

$$\text{ΔCD}4(\text{t})=\text{max}(\text{ΔCD}4_{0}+\text{αt},\;-\text{CD}4_{\text{ref}}(\text{t})).$$

This model (Equation 1) is applied to both the CD4ct and the CD4% data. We estimated ∆CD4_0_, *α*, and T_A_ by minimizing the sum of squared residuals between the model and the data for every individual child. We use therefore the R (version 4.0.5) package FME with its default Levenberg–Marquardt fitting algorithm.^20^ We determined lower and upper boundaries for parameter estimates: ∆CD4_0_ ∈ [−CD4_ref_ (0), CD4_ref_ (0)], *α*_ct_ ∈ [−50, 50] for CD4ct, *α*_%_ ∈ [−1, 1] for CD4%, and T_A_ ∈ [0, t_end_]. Thus, these boundaries can vary per individual. For ∆CD4_0_, the boundaries were chosen to ensure that the initial values result in positive T-cell numbers and do not exceed a doubling of the healthy reference value. The boundaries for the recovery rates, *α*, are arbitrarily chosen. Using the time of the last observation (t_end_) as an upper bound for T_A_ may have shortened the actual time to reach an asymptote and lead to a lower ∆CD4_A_, but allowing for later estimates for T_A_ would have been arbitrary (as no later data are available). ∆CD4_A_ is not a free parameter and was derived from the model (Equation 1).

To exclude children with treatment complications, we first only considered children with a monotonically declining VL to viral suppression (N = 188). To be able to study the early CD4 dynamics after ART initiation, we required at least 5 measurements, of which at least 2 had to be collected within the first 120 days of ART for each child (N_ct_ = 119, N_%_ = 117). If there was no baseline measurement available at time t_0_, we extrapolated baseline values from the closest measurements up to 10 days before ART initiation. We only considered those fits where the modFit function of the FME package was able to compute the Hessian matrix because standard errors for all 3 parameter estimates were provided for these fits. We considered parameters to be identifiable when all parameter estimates were significantly different from zero, to a significance level of 0.05 according to the t-statistic. Finally, we required at least the first 2 measurements before T_A_ because otherwise the recovery rate cannot be estimated.

## RESULTS

### Early ART Initiation Leads to CD4 Reconstitution

**FIGURE 2.**
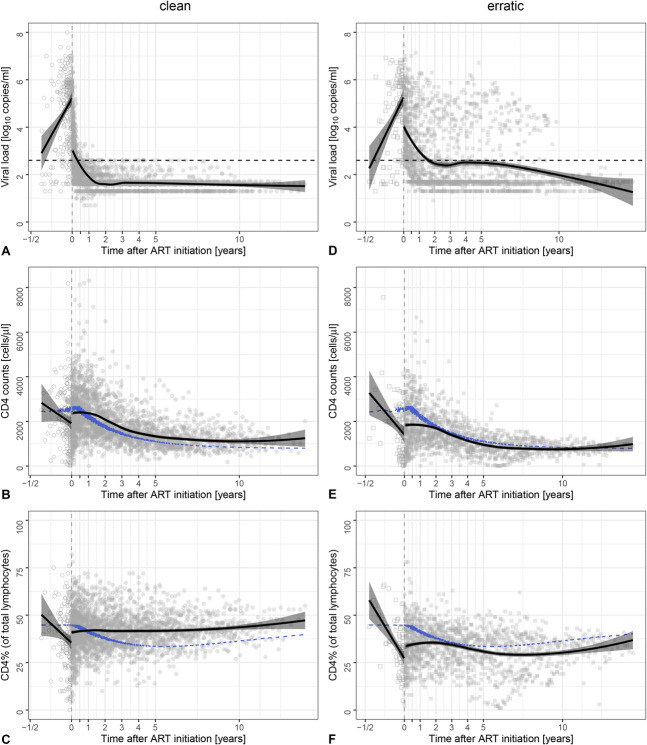
Reconstitution of CD4 levels after early ART initiation. Cross-sectional presentation of the trajectories of the VL (A, D), CD4^+^ T-cell count (B, E), and CD4^+^ T-cell percentage (C, F) after ART initiation in 188 infants who suppressed their VL in a clean manner (left panels A–C, circles), and from 88 infants who suppressed their VL erratically (right panels D–F, squares). Each symbol represents an individual measurement (with multiple measurements per individual). Open symbols are measurements before ART initiation, and filled symbols are measurements during treatment. The vertical gray dashed lines indicate the start of ART at time zero. Black solid lines present the regression lines with their 95% confidence intervals. For the measurements before ART initiation, linear regression was performed; for the measurements after ART initiation, the locally estimated scatter plot smoothing (LOESS) regression lines are depicted. The horizontal black dashed lines in (A, D) present the VL threshold value of log_10_ (400 copies/mL). Black dots in (B), (C), (E), and (F) represent median age-matched healthy reference values, retrieved from Schröter et al.^13^

We investigated CD4^+^ T-cell reconstitution in virally suppressed infants (N = 276, see Table S1, Supplemental Digital Content, http://links.lww.com/QAI/B793), who acquired HIV-1 perinatally and started standard ART within median age of 82 days [IQR = (34, 121)]. These infants achieved viral suppression after a median of 131 days [IQR = (63, 282); 1 day earlier than described in Schröter et al^16^ because we added 1 day to be able to plot time to viral suppression on a logarithmic scale and previously did not correct for this in the summary statistics] and remained virally suppressed for a median of 836 days [IQR = (333, 2108), Figs. 2A, D] while on treatment. Previously, we described that viral suppression can be achieved in a “clean” manner, with a VL declining monotonically (N = 188, Fig. 2A) or in an “erratic” manner, with VL measurements that also increase before viral suppression is attained (N = 88, Fig. 2D).^16^ Because the latter might be resulting from treatment complications, we first focus on children with “clean” viral suppression patterns after treatment initiation.

First, we computed cross-sectional CD4 trajectories to depict the median trend of CD4^+^ T-cell reconstitution after ART initiation (Figs. 2B, C, see Fig. S1A, B, Supplemental Digital Content, http://links.lww.com/QAI/B786). Before treatment initiation, both the CD4ct and the CD4% decline. After ART initiation, the CD4ct (Fig. 2B and see Fig. S1A, Supplemental Digital Content, http://links.lww.com/QAI/B786) and CD4% (Fig. 2C and see Fig. S1B, Supplemental Digital Content, http://links.lww.com/QAI/B786) stopped decreasing, stabilized, started increasing, and approached values exceeding the median healthy reference value somewhat. Most children's CD4 levels (referring to both the CD4ct and the CD4%, Figs. 2B, C, see Fig. S1A, B, Supplemental Digital Content, http://links.lww.com/QAI/B786) subsequently followed the natural CD4 trajectory. These cross-sectional CD4 trajectories suggest that CD4 levels reach suprahealthy levels under ART. The CD4% (Fig. 2C, see Fig. S1B, Supplemental Digital Content, http://links.lww.com/QAI/B786) takes somewhat longer to stabilize than the CD4ct (Fig. 2B, see Fig. S1A, Supplemental Digital Content, http://links.lww.com/QAI/B786). In the following sections, we quantify CD4 recovery by modeling individual CD4 trajectories longitudinally.

### Quantification of the CD4ct Recovery

**FIGURE 3.**
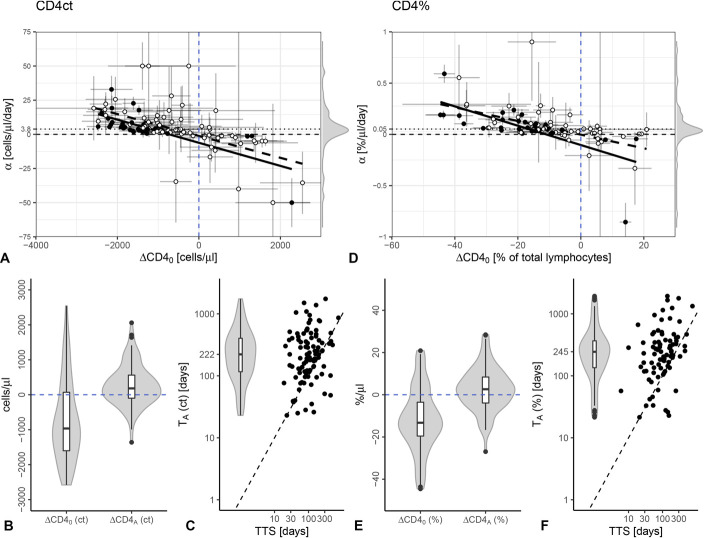
Similar CD4 recovery dynamics of the CD4ct and CD4%. Panels (A–F) summarize the model estimates. The results for the CD4ct are represented on the left side (A–C), and those for the CD4% are represented on the right side (D–F). In panels (A, D), the parameter estimates of ΔCD4_0_ are plotted against the parameter estimates of *α*. Each symbol shows the combination for 1 individual. Filled circles reflect that the estimates for all 3 parameters (CD4_0_, *α*, and T_A_) were identifiable. The standard errors are depicted for both estimates by the error bars, for ΔCD4_0_ in horizontal and for *α* in vertical directions. The dashed lines represent the regression lines for all accepted estimates, and the black regression lines are drawn only for fits where all 3 parameters were identifiable. A density plot of the parameter estimates for *α* is illustrated in the margin of the panels. The median recovery rate values are indicated by a horizontal dotted lines. In panels (B, E), the distributions of ΔCD4_0_ and ΔCD4_A_ are shown by a violin plots with integrated boxplots. The healthy reference CD4 levels are depicted by the black dashed lines at zero. In (C, F), the distribution of T_A_ (violin plot) and its correlations with time to viral suppression (TTS) are illustrated. The dashed black lines represent the identity lines.

First, we constructed individual reconstitution trajectories of the CD4ct. To be able to compare CD4 trajectories between infants, we took the natural CD4 decline into account and computed CD4 measurements relative to their age-matched reference values (∆CD4, Fig. 1 and see Fig. S1, Supplemental Digital Content, http://links.lww.com/QAI/B786). Next, we fitted the ∆CD4ct data per infant using the mathematical model described in Equation (1) and obtained reliable CD4ct fits for 97 infants (for 32 fits, all parameters, CD4_0_, *α*, and T_A_, were identifiable) (Figs. 3A–C, see Fig. S2, Supplemental Digital Content, http://links.lww.com/QAI/B787). At the start of treatment, the median CD4ct was below the median healthy age-matched level [∆CD4_0_ = −965 cells/*µL*, IQR = (−1602, 71)], but a quarter of the infants maintained their CD4ct above median healthy age-matched values (25 of 97 infants, Fig. 3A).^13^ During ART, the CD4ct increased by a median of 3.8 cells/*µL*/d [IQR = (0.5, 9.6), Fig. 3A], and 64 infants acquired median healthy values [median ∆CD4_A_ = 182 cells/*µL*, IQR = (−98, 562); Fig. 3B]. The reconstitution rate *α*_ct_ was negatively associated with the CD4ct at start of treatment (Spearman correlation: *ρ* = −0.67, *P*-value < 0.001, Fig. 3A), indicating that the recovery rate increases when cell numbers are low. Infants stabilized their CD4ct within a median of 222 days [IQR = (116, 403), Fig. 3C]. Note that the estimated time to stabilize CD4ct remains a lower bound because not every child reached a stable CD4ct by the end of the observation period. In the majority of cases (N = 81), CD4ct recovery (T_A_) took longer than viral suppression (time to viral suppression was defined as the time to the first of 2 consecutive VL measurements of <400 copies/mL^16^), which took a median of 97 days [IQR = (58, 168), Fig. 3C]. The time to stabilize the CD4ct has a weak positive association with the time to viral suppression (Spearman correlation: *ρ* = 0.21, *P*-value = 0.04, Fig. 3C). Previously, we showed that slower viral suppression is associated with high initial VL and low CD4 levels, both of which reflect HIV disease progression status.^16^ Thus, further progression of HIV infection results not only in a lower ∆CD4ct_0_ but also in a longer time to recover. We noticed that not all infants increased their ∆CD4ct and that 23 experienced a further decline in their CD4ct despite being on ART (Fig. 3A).

However, the majority of these infants (N = 21) had a supranormal CD4ct, that is, a positive ∆CD4ct, so it is unsurprising that their CD4ct decreased rather than increased. As the parameter estimates for infants with declining ∆CD4ct were mostly not significant (N = 20, Fig. 3A), we will not address them further. Summarizing, after treatment initiation, the CD4ct recovers at a rate of 3.8 cells/*µL*/d and within approximately 200 days.

### Quantification of the CD4% Recovery

Second, we investigated the dynamics of CD4% within individuals after treatment initiation, again relative to the median age-matched healthy values. We obtained ∆CD4% fits for 86 infants, and for 34 children, all parameters were identifiable (Fig. 3D, see Fig. S2, Supplemental Digital Content, http://links.lww.com/QAI/B787). Infants started ART with a median ∆CD4% of −13.27% [IQR = (−19.56, −3.53), Fig. 3D], and reconstitution occurred at a median rate of 0.05%/day [IQR = (0.02, 0.12), Fig. 3D]. CD4% reconstitution rates and the initial ∆CD4_0_% were negatively correlated (Spearman *ρ* = −0.67, *P* < 0.001, Fig. 3D), suggesting a density dependence in CD4 reconstitution, such that more profound depletion in CD4^+^ T cells induces more rapid rebound. The CD4% stabilized at a median of 245 days [IQR = (135, 375), Fig. 3F]. A stable CD4% is again reached after viral suppression occurred after a median of 106 days [IQR = (57, 168), Fig. 3F]. The correlation between the time to CD4% stabilization and the time to viral suppression were comparable with those of the CD4ct (Spearman *ρ* = 0.36, *P* < 0.001, Figs. 3C, F). The ∆CD4% asymptote levels differed by a median of 2.61% [IQR = (−3.91, 8.40), Fig. 3E], implying that the majority of infants (N = 55) exceeded median healthy values. Thus, the CD4% recovery is comparable with the CD4ct recovery.

### The TLC Also Increase With Treatment Initiation

**FIGURE 4.**
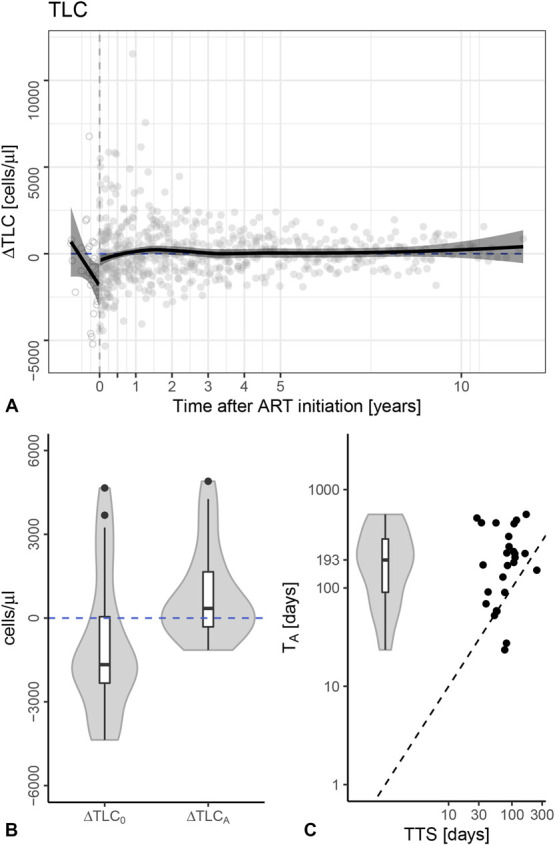
Dynamics of total lymphocyte count (TLC). Panel (A) depicts cross-sectional trajectories of age-matched ∆TLC trajectories of a subset of 57 infants from the CHIPS cohort (an EPPICC subset). Each symbol represents the difference of a measurement to age-matched median healthy reference value (∆TLC). Open symbols are measurements before the initiation of ART, and filled symbols are measurements after ART initiation. The vertical gray dashed lines indicate the start of ART at time zero. The solid black lines present regression lines with their 95% confidence intervals. For the measurements before ART initiation, a linear regression is performed; for the measurements after ART initiation, the LOESS regression line is depicted. The horizontal black dashed lines at zero represent the median healthy reference values, retrieved from Schröter et al.^13^ In panel (B), the distributions of the initial differences and the differences of the asymptote levels are shown by a violin plots with integrated boxplots, ∆TLC_0_ and ∆TLC_A_. The healthy reference TLC levels are depicted by the black dashed lines at zero. Panel (C) depicts the distribution of T_A_ (violin plot) and its correlation with time to viral suppression (TTS). The dashed black lines represent the identity lines.

TLC data were available for 58 infants belonging to the CHIPS cohort within EPPICC. We investigated the TLC dynamics of 33 infants fulfilling the criteria of “clean” viral suppression (Fig. 4, see Fig. S3, S4, Supplemental Digital Content, http://links.lww.com/QAI/B790, http://links.lww.com/QAI/B791). Cross-sectional trajectories suggest that TLC and CD4ct exhibit similar patterns of reconstitution after ART initiation (Fig. 4A). We quantified TLC reconstitution by fitting individual ∆TLC trajectories using our model Equation (1) and obtained 26 fits (Figs. 4B, C, see Fig. S3, S4, Supplemental Digital Content, http://links.lww.com/QAI/B790, http://links.lww.com/QAI/B791). The TLC increased initially by a median of 8.5 cells/*µL*/d [IQR = (−0.1, 18.9), see Fig. S4, Supplemental Digital Content, http://links.lww.com/QAI/B791], which overlaps with the rate at which the CD4ct recover. The times to stabilization [T_A_ (TLC) = 193 days, IQR = (90, 316), Fig. 4C] are in the same range as those of the CD4ct, and although the initial TLC [∆TLC_0_ = −1670, IQR = (−2330, 48), see Fig. S4, Supplemental Digital Content, http://links.lww.com/QAI/B791] is markedly reduced, the range overlaps with that of the CD4ct. The median stable level (∆TLC_A_) with 345 cells/*µL* [IQR = (−315, 1655)] is also somewhat above healthy TLC levels (Fig. 4B). Thus, the TLC and CD4ct recovery show comparable recovery dynamics, which is not unexpected because the depletion of the TLC during HIV infection should largely be because of a depletion of the CD4^+^ T-cell counts.

### CD4 Recovery Is Density-Dependent Rather Than Age-Dependent

According to the current literature, the timing and the levels of CD4 reconstitution depend on age at the start of treatment and on the initial CD4 levels.^4–6^ We found evidence for an increase in the rate of CD4ct recovery when the CD4^+^ T-cell density is low. Because CD4 levels decline before treatment (Figs. 2B–F), we then expect age to be positively associated with the recovery rate. We confirmed this by studying the impact of the age at start of treatment on the dynamics of CD4 reconstitution. The individual initial CD4ct (∆CD4ct_0_, Spearman *ρ* = −0.35, *P* < 0.001, see Fig. S5A, Supplemental Digital Content, http://links.lww.com/QAI/B792) and CD4 recovery rate (Spearman *ρ* = 0.30, *P* = 0.002, see Fig. S5B, Supplemental Digital Content, http://links.lww.com/QAI/B792) were weakly correlated with age at start of treatment. The time to stabilize and the CD4 recovery levels (CD4_A_), however, do not show any significant correlations with age at start of treatment (see Fig. S5C, D, Supplemental Digital Content, http://links.lww.com/QAI/B792). Thus, we find no evidence for an age-dependent CD4ct reconstitution in these individuals. We find similar results for the reconstitution of the CD4%. Although the initial ∆CD4% declines with age (Spearman *ρ* = −0.45, *P* < 10^−4^; see Fig. S5E, Supplemental Digital Content, http://links.lww.com/QAI/B792) and the CD4% recovery rate (Spearman correlation: *ρ* = 0.37, *P*-value < 0.001; see Fig. S5F, Supplemental Digital Content, http://links.lww.com/QAI/B792) correlates with age, the asymptote level and time to stabilize are independent of the age at start of treatment. The positive effect of age on the recovery rate could therefore be indirect and because of a density-dependent recovery of the CD4%. Summarizing, we find no evidence for a direct effect of age on CD4^+^ T-cell reconstitution in these early-treated children.

### CD4 Recovery Is Delayed in Infants With Erratic Viral Suppression Patterns

Finally, we return to the children with an erratic viral suppression pattern (N = 88, Figs. 2D–F). Given that CD4 recovery occurs in children experiencing a monotonic decline of VL on ART, it is interesting to explore whether perinatally HIV-acquired children with treatment complications, and/or erratic viral suppression patterns, also experience a “full” recovery of their immune system. Because CD4 trajectories of “erratic” children are difficult to model, we interpreted their cross-sectional data visually (Figs. 2D–F and see Fig. S1C, D, Supplemental Digital Content, http://links.lww.com/QAI/B786). Infants with erratic viral suppression patterns seem to exhibit a slower CD4 recovery than infants in the “clean” subset (Fig. 2, see Fig. S1, Supplemental Digital Content, http://links.lww.com/QAI/B786). Treatment halts the decline of CD4^+^ T-cell levels, and reconstitution tends to stabilize only after the VL is fully suppressed. Ultimately, the CD4ct and CD4% do approach median healthy levels (Figs. 2E, F, see Fig. S1C, D, Supplemental Digital Content, http://links.lww.com/QAI/B786). Infants with erratic viral suppression patterns experience significantly longer times to viral suppression than infants with clean viral suppression patterns [clean: 90 days (48, 166), erratic: 375 days (204, 855)],^16^ which might explain the delay in CD4 recovery. The asymptotic CD4 levels tend to be lower in “erratic” infants than in “clean” ones (Figs. 2C, F, see Fig. S1B, D, Supplemental Digital Content, http://links.lww.com/QAI/B786). The lower initial CD4 levels in the erratic subset suggest that the infection has progressed further in the pretreatment phase. The slower recovery in the erratic subset is probably also because of a longer viral exposure during treatment, since prolonged immune activation may delay the reconstitution of CD4^+^ T cells. Thus, early initiation of ART in infants reduces HIV disease progression and enables reconstitution of CD4^+^ T cells despite erratic viral suppression.

## DISCUSSION

Early initiation of ART in individuals living with HIV has been proven to be very effective in stabilizing and reconstituting CD4 levels and to prevent progression to AIDS, and this is particularly true in children.^1,5,10,21–23^ Here, we quantified the trajectories of CD4^+^ T-cell recovery in very-early treated, perinatally HIV-acquired infants using mathematical modeling. Depleted CD4ct generally increase by approximately 4 cells/*µL*/d in infants and recover to healthy age-matched levels in about 3–13 months. These estimates are in line with other pediatric studies.^2,24^ The estimated recovery rate in infants is about 3-fold faster than those reported values for adults.^2,24–28^ For example, Hardy et al^27^ report an increase of 147 cells/*µL* within 16 weeks in adults on ART, corresponding to a recovery rate of 1.3 cells/*µL*/d.

The CD4^+^ T-cell reconstitution process can be divided into 2 phases: (1) early redistribution of T cells from lymphoid tissue and (2) de novo production of T cells by peripheral cell division and/or the thymus.^25,29,30^ Our model (Equation 1) unifies the 2 phases into a single linear increase because we typically have no data to capture the early redistribution of T cells (the second data point is typically after 1 month). In adults, redistribution generally occurs within the first month of treatment,^26,31,32^ which is much shorter than our times of follow-up and our estimated times to recover. Thus, the linear recovery that we model largely captures the second phase. If the true recovery were nonlinear because of an additional early redistribution, our model with a single linear recovery phase would have overestimated the initial CD4_0_ values and have averaged the recovery rates. However, we expect these effects to be minor and conclude that a model with a linear increase is an appropriate conservative choice.

Our estimated recovery rate of 4 cells/*µL*/d in young children is fairly slow. The daily production of naive T cells by the thymus in children has been estimated in 2 articles.^28,33^ For a 2-year-old child, which is a midpoint of most of our recovery phases, the estimates for the total body production vary from 0.3 × 10^9^ cells^28^ to 1.3 × 10^9^ cells^33^ per day. In the same articles, the blood volume of a 2-year-old child is estimated to be about a 1000 mL. Assuming that 2% of the T cells reside in the blood, and recalculating into microliters, these published estimates vary from 6 to 26 cells/*µL*/d, which is the same range, but somewhat higher than our median estimate. Because our estimated recovery rate of 4 cells/*µL*/d should also include production of memory CD4^+^ T cells, this suggest the CD4 recovery in children living HIV is slower than the normal daily production would allow for.

A monophasic model of reconstitution, in combination with a normalization of CD4^+^ T-cell levels to age-matched reference values, is commonly used for modeling pediatric CD4 recovery.^4–6,24^ We nevertheless introduced few improvements. First, for the normalization, we do not use the conventional z-scores,^34^ but we express our data as the distance from the age-matched reference value (ie, zero is the normal value). This choice allows for a more intuitive graphical representation of the data and for a more intuitive interpretation of the estimated parameters. For instance, the units of the recovery rate are given in cells per microliter per day. Second, we use a linear model approaching an asymptote rather than an exponential model with an asymptote.^5,6^ The number of estimated parameters is the same, but we prefer the linear model of our Equation (1) because (1) it is less sensitive to a potential initial increase because of redistribution; and (2) it naturally identifies the time at which CD4 levels stabilize. Finally, in contrast to the two-knot linear spline model used by Simms et al,^4^ we do not fix the time at which CD4 levels stabilize, but estimate it. Although Simms et al^4^ considered the first 3 months after ART initiation as the main window for CD4 reconstitution in children and fixed the time of CD4 stabilization to 84 days, we find in our data that CD4 reconstitution is considerably slower with both CD4ct and CD4% stabilizing around 200 days after ART initiation. We find that the time to CD4 recovery is at least 100 days later than the time to viral suppression, which is in agreement with previous studies associating CD4 recovery with viral exposure time.^35,36^ For a few patients with a short follow-up, our time to stabilization should be taken as a lower bound because the long-term asymptote may not be reached within the observation window. Summarizing, we present here a new quantitative framework for describing CD4 recovery dynamics in infants.

Our analyses suggest that the rate of CD4^+^ T-cell recovery increases when cell densities are low, which is in good agreement with previous studies showing that CD4 recovery is density-dependent.^5,6,35^ However, these results should be interpreted with care because they could be because of a regression to the mean effect, that is, a too low or too high estimate of the initial CD4 level will lead to a too high or too low estimate of the recovery rate at the next time point. In addition, we show that the asymptote levels and the time to stabilize are all independent of age in these very-early treated infants. Van Rossum et al^24^ and Vrisekoop et al^37^ also argue that CD4^+^ T-cell recovery is age-independent. The contribution of age on CD4^+^ T-cell recovery is extensively discussed in the literature and might play a role in older-treated children.^22,35,38^ In this cohort of very young children, the most rapid recovery of the immune system is achieved with early and successful viral suppression, which can be achieved by early treatment initiation.

In the field of immunology, it is often debated whether the CD4ct or CD4% should be used.^39^ Because the CD4ct are much more sensitive to measurement errors, the CD4% was generally recommended as a biomarker for HIV disease progression and as an indicator to initiate treatment in infants.^40^ However, the majority of CD4 recovery models are based on CD4ct data. We have modeled both and have shown similar general recovery dynamics for age-normalized CD4ct and CD4%. Summarizing, considering CD4ct and CD4% data together is most informative because the CD4ct is more noisy but better reflects reconstitution levels, and the CD4% is less variable but is also influenced by other components of the cellular immune system.

In conclusion, early-treated children achieving sustained viral suppression typically attain “healthy” CD4 levels. This is the remarkable achievement of ART, allowing HIV-infected people to live a long and “almost healthy” life.^9,29^ However, perinatally HIV-acquired infants carry the high burden of being on life-long treatment, affecting their general (immuno-, neuro-) development and might lead to yet unknown side effects.^1,41,42^ Moreover, long-term drug use and stigmatization can result in psychological difficulties once these children enter adolescence.^43^ Current pediatric HIV research therefore focuses on providing simpler, more tolerable regimens, faster, and novel strategies such as weekends off, which makes ART easier to take.^1,12,44,45^ Controlled treatment interruption is sometimes successful in children and has resulted in the maintenance of viral suppression off-treatment.^7,8,46^ However, the optimal timing and medical conditions for scheduled treatment interruptions or additional immunotherapies next to ART remain elusive. Quantification of the CD4 recovery and the viral suppression, such as we provide here, can inform the timing of such an intervention.

## APPENDIX

The EPPICC cohorts:

**Belgium:** Hospital St Pierre Cohort, Brussels: Tessa Goetghebuer, MD, PhD; Marc Hainaut, MD, PhD; Evelyne Van der Kelen, Research nurse; Marc Delforge, data manager.

**France:** French Perinatal Cohort Study/Enquê te Périnatale Française, ANRS EPF-CO10. Coordinating center, INSERM U1018, team 4: Josiane Warszawski, Jerome Le Chenadec, Elisa Ramos, Olivia Dialla, Thierry Wack, Corine Laurent, Lamya Ait si Selmi, Isabelle Leymarie, Fazia Ait Benali, Maud Brossard, and Leila Boufassa.

*Participating sites (hospital name, city, main investigator):* Hôpital Louis Mourier, Colombes, Dr. Corinne Floch-Tudal; Groupe Hospitalier Cochin Tarnier Port-Royal, PARIS, Dr. Ghislaine Firtion; Centre Hospitalier Intercommunal, Creteil, Dr. Isabelle Hau; Centre Hospitalier Général, Villeneuve Saint Georges, Dr. Anne Chace; Centre Hospitalier Général-Hôpital Delafontaine, Saint-Denis, Dr. Pascal Bolot; Groupe Hospitalier Necker, Paris, Pr Stéphane Blanche; Centre hospitalier Francilien Sud, Corbeil Essonne, Dr. Michéle Granier; Hôpital Antoine Béclére, Clamart, Pr Philippe Labrune; Hôpital Jean Verdier, Bondy, Dr. Eric Lachassine; Hôpital Trousseau, Paris, Dr. Catherine Dollfus; Hôpital Robert Debré, Paris, Dr. Martine Levine; Hôpital Bicêtre, Le Kremlin Bicëtre, Dr. Corinne Fourcade; Centre Hospitalier Intercommunal, Montreuil, Dr. Brigitte Heller-Roussin; Centre Hospitalier Pellegrin, Bordeaux, Dr. Camille Runel-Belliard; CHU Paule de Viguier, Toulouse, Dr. Joëlle Tricoire; CHU Hôpital de l’Archet II, Nice, Dr. Fabrice Monpoux; Groupe Hospitalier de la Timone, Marseille; CHU Hôpital Jean Minjoz, Besancon, Dr. Catherine Chirouze; CHU Nantes Hotel Dieu, Nantes, Dr. Véronique Reliquet; CHU Caen, Caen, Pr Jacques Brouard; Institut d’Hématologie et Oncologie Pédiatrique, Lyon, Dr. Kamila Kebaili; CHU Angers, Angers, Dr. Pascale Fialaire; CHR Arnaud de Villeneuve, Montpellier, Dr. Muriel Lalande; CHR Jeanne de Flandres, Lille, Dr. Françoise Mazingue; Hôpital Civil, Strasbourg, and Dr. Maria Luisa Partisani.

**Germany:** German Paediatric & Adolescent HIV Cohort (GEPIC): Dr. Christoph Königs and Dr. Stephan Schultze-Strasser. *German clinical centers:* Hannover Medical School, Dr. U. Baumann; Pediatric Hospital Krefeld, Dr. T. Niehues; University Hospital Düsseldorf, Dr. J. Neubert; University Hospital Hamburg, Dr. R. Kobbe; Charite Berlin, Dr. C. Feiterna-Sperling; University Hospital Frankfurt, Dr. C. Königs; University Hospital Mannheim, Dr. B. Buchholz; Munich University Hospital, Dr. G. Notheis.

**Greece:** Greek cohort: Vana Spoulou.

**Italy:** Italian Register for HIV infection in Children. Coordinators: Maurizio de Martino (Florence), Pier Angelo Tovo (Turin). Participants: Osimani Patrizia (Ancona), Domenico Larovere (Bari), Maurizio Ruggeri (Bergamo), Giacomo Faldella, Francesco Baldi (Bologna) Raffaele Badolato (Brescia), Carlotta Montagnani, Elisabetta Venturini, Catiuscia Lisi (Florence), Antonio Di Biagio, Lucia Taramasso (Genua), Vania Giacomet, Paola Erba, Susanna Esposito, Rita Lipreri, Filippo Salvini, Claudia Tagliabue (Milan), Monica Cellini (Modena), Eugenia Bruzzese, Andrea Lo Vecchio (Naples), Osvalda Rampon, Daniele Donà (Padua), Amelia Romano (Palermo), Icilio Dodi (Parma), Anna Maccabruni (Pavia), Rita Consolini (Pisa), Stefania Bernardi, Hyppolite Tchidjou Kuekou, Orazio Genovese (Rome), Paolina Olmeo (Sassari), Letizia Cristiano (Taranto), Antonio Mazza (Trento), Clara Gabiano, Silvia Garazzino (Turin), and Antonio Pellegatta (Varese).

**Netherlands:** The ATHENA database is maintained by Stichting HIV Monitoring and supported by a grant from the Dutch Ministry of Health, Welfare and Sport through the Centre for Infectious Disease Control of the National Institute for Public Health and the Environment.

Clinical centers (Paediatric care): Emma Kinderziekenhuis, Amsterdam Universitair Medische Centra: HIV treating physicians: D. Pajkrt, H. J. Scherpbier; HIV nurse consultants: C. de Boer, A. M. Weijsenfeld; HIV clinical virologists/chemists: S. Jurriaans, N. K. T. Back, H. L. Zaaijer, B. Berkhout, M. T. E. Cornelissen, C. J. Schinkel, K. C. Wolthers. Erasmus MC–Sophia, Rotterdam: HIV treating physicians: P. L. A. Fraaij, A. M. C. van Rossum, C. L. Vermont; HIV nurse consultants: L. C. van der Knaap, E. G. Visser; HIV clinical virologists/chemists: C. A. B. Boucher, M. P. G. Koopmans, J. J. A van Kampen, S. D. Pas. Radboudumc, Nijmegen: HIV treating physicians: S. S. V. Henriet, *M. van* de Flier, K. van Aerde; HIV nurse consultants: R. Strik-Albers; HIV clinical virologists/chemists: J. Rahamat-Langendoen, F. F. Stelma. Universitair Medisch Centrum Groningen, Groningen: HIV treating physicians: E. H. Schölvinck; HIV nurse consultants: H. de Groot-de Jonge; HIV clinical virologists/chemists: H. G. M. Niesters, C. C. van Leer-Buter, M. Knoester. Wilhelmina Kinderziekenhuis, UMC Utrecht, Utrecht: HIV treating physicians: L. J. Bont, S. P. M. Geelen, T. F. W. Wolfs; HIV nurse consultants: N. Nauta; HIV clinical virologists/chemists: R. Schuurman, F. Verduyn-Lunel, and A. M. J. Wensing.

Coordinating Center: Director: P. Reiss; Data analysis: D. O. Bezemer, A. I. van Sighem, C. Smit, F. W. M. N. Wit, and T. S. Boender; Data management and quality control: S. Zaheri, M. Hillebregt, and A. de Jong; Data monitoring: D. Bergsma, S. Grivell, A. Jansen, M. Raethke, and R. Meijering; Data collection: L. de Groot, M. van den Akker, Y. Bakker, E. Claessen, A. El Berkaoui, J. Koops, E. Kruijne, C. Lodewijk, L. Munjishvili, B. Peeck, C. Ree, R. Regtop, Y. Ruijs, T. Rutkens, M. Schoorl, A. Tim-merman, E. Tuijn, L. Veenenberg, S. van der Vliet, A. Wisse, and T. Woudstra; Patient registration: B. Tuk.

**Poland:** Polish pediatric cohort: Head of the team: Prof Magdalena Marczyńska, MD, PhD. Members of the team: Jolanta Popielska, MD, PhD; Maria Pokorska-Śpiewak, MD, PhD; Agnieszka Ołdakowska, MD, PhD; Konrad Zawadka, MD, PhD; Urszula Coupland, MD, PhD. Administration assistant: Małgorzata Doroba. Affiliation: Medical University of Warsaw, Poland, Department of Children's Infectious Diseases; Hospital of Infectious Diseases in Warsaw, Poland.

**Portugal:** Centro Hospitalar do Porto: Laura Marques, Carla Teixeira, Alexandre Fernandes. Departamento de Pediatria/Hospital de Santa Maria/CHULN: Filipa Prata.

**Romania:** “Victor Babes” Hospital Cohort, Bucharest: Dr. Luminita Ene.

**Russia:** Federal State-owned Institution “Republican Clinical Infectious Diseases Hospital” of the Ministry of Health of the Russian Federation, St Petersburg: Liubov Okhonskaia, Evgeny Voronin, Milana Miloenko, Svetlana Labutina.

**Spain:** CoRISPE-cat, Catalonia: Financial support for CoRISPE-cat was provided by the Instituto de Salud Carlos III through the Red Temática de Investigación Cooperativa en Sida. Members: Hospital Universitari Vall d’Hebron, Barcelona (Pere Soler-Palacín, Maria Antoinette Frick and Santiago Pérez-Hoyos (statistician)), Hospital Universitari del Mar, Barcelona (Antonio Mur, Núria López), Hospital Universitari Germans Trias i Pujol, Badalona (María Méndez), Hospital Universitari JosepTrueta, Girona (Lluís Mayol), Hospital Universitari Arnau de Vilanova, Lleida (Teresa Vallmanya), Hospital Universitari Joan XXIII, Tarragona (Olga Calavia), Consorci Sanitari del Maresme, Mataró (Lourdes García), Hospital General de Granollers (Maite Coll), Corporació Sanitària Parc Taulí, Sabadell (Valentí Pineda), Hospital Universitari Sant Joan, Reus (Neus Rius), Fundació Althaia, Manresa (Nu’ ria Rovira), Hospital Son Espases, Mallorca (Joaquín Dueñas) and Hospital Sant Joan de Déu, Esplugues (Cláudia Fortuny, Antoni Noguera-Julian).

CoRISPE and Madrid Cohort Working Group: María José Mellad o, Luis Escosa, Milagros García Hortelano, Talía Sainz (Hospital Universitario La Paz, Madrid); Pablo Rojo, Daniel Blázquez, Luis Prieto-Tato, Cristina Epalza (Hospital Universitario Doce de Octubre, Madrid); José Tomás Ramos (Hospital Clínico San Carlos, Madrid); Sara Guillén (Hospital Universitario de Getafe, Madrid); María Luisa Navarro, Jesús Saavedra, Mar Santos, Begoña Santiago, Santiago Jimenez de Ory, Itzíar Carrasco, Ma Angeles Muñoz-Fernández (Hospital Universitario Gregorio Marañón, Madrid); Miguel Ángel Roa (Hospital Universitario de Móstoles, Madrid); María Penín (Hospital Universitario Príncipe de Asturias de Alcalá de Henares, Madrid); Jorge Martínez (Hospital Infantil Universitario Niño Jesús, Madrid); Katie Badillo (Hospital Universitario de Torrejón, Madrid); Eider Oñate (Hospital Universitario Donostia, Guipúzcoa); Itziar Pocheville (Hospital Universitario Cruces, Vizcaya); Elisa Garrote (Hospital Universitario Basurto, Vizcaya); Elena Colino (Hospital Insular Materno Infantil, Gran Canaria); Jorge Gómez Sirvent (Hospital Universitario Virgen de la Candelaria, Tenerife); Mónica Garzón, Vicente Román (Hospital General, Lanzarote); Raquel Angulo (Hospital de Poniente de El Ejido, Almería); Olaf Neth, Lola Falcón (Hospital Universitario Virgen del Rocío, Sevilla); Pedro Terol (Hospital Universitario Virgen de la Macarena, Sevilla); Juan Luis Santos (Hospital Universitario Virgen de las Nieves, Granada); David Moreno (Hospital Regional Universitario Carlos Haya, Málaga); Francisco Lendínez (Complejo Hospitalario Torrecárdenas, Almería); Estrella Peromingo (Hospital Universitario Puerta del Mar, Cádiz); José Uberos (Hospital Clínico San Cecilio, Granada); Beatriz Ruiz (Hospital Universitario Reina Sofía de Córdoba); Ana Grande (Complejo Hospitalario Universitario Infanta Cristina, Badajoz); Francisco José Romero (Complejo Hospitalario, Cáceres); Carlos Pérez (Hospital de Cabueñes, Asturias); Miguel Lillo (Complejo Hospitalario Universitario, Albacete); Begoña Losada (Hospital Virgen de la Salud, Toledo); Mercedes Herranz (Hospital Virgen del Camino, Navarra); Matilde Bustillo (Hospital Universitario Miguel Servet, Zaragoza); Pilar Collado (Hospital Clínico Universitario Lozano Blesa, Zaragoza); José Antonio Couceiro (Complejo Hospitalario Universitario, Pontevedra); Leticia Vila (Complejo Hospitalario Universitario, La Coruña); Consuelo Calviño (Hospital Universitario Lucus Augusti, Lugo); Ana Isabel Piqueras, Manuel Oltra (Hospital Universitario La Fe, Valencia); Cé sar Gavilán (Hospital Universitario de San Juan de Alicante, Alicante); Elena Montesinos (Hospital General Universitario, Valencia); Marta Dapena (Hospital General, Castellón); Cristina Á lvarez, Beatriz Jimé nez (Hospital Universitario Marqués de Valdecilla, Cantabria); Ana Gloria Andrés (Complejo Hospitalario, León); Víctor Marugán, Carlos Ochoa (Complejo Hospitalario, Zamora); Santiago Alfayate, Ana Isabel Menasalvas (Hospital Universitario Virgen de la Arrixaca, Murcia); Yolanda Ruiz del Prado (Complejo Hospitalario San Millán-San Pedro, la Rioja) and Paediatric HIV-BioBank integrated in the Spanish AIDS Research Network and collaborating Centers.

Financial support for CoRISpeS and Madrid Cohort was provided by the Instituto de Salud Carlos III through the Red Tematica de Investigacion Cooperativa en Sida (RED-RIS) project as part of the Plan R + D + I and cofinanced by ISCIII-Subdireccion General de Evaluación and Fondo Europeo de Desarrollo Regional (FEDER).

**Sweden:** Karolinska Institutet and University Hospital, Stockholm (Lars Naver, Sandra Soeria-Atmadja, Vendela Hagås).

**Switzerland:** Members of the Swiss HIV Cohort Study (SHCS) and the Swiss Mother and Child HIV Cohort Study: Aebi-Popp K, Anagnostopoulos A, Battegay M, Baumann M, Bernasconi E, Böni J, Braun DL, Bucher HC, Calmy A, Cavassini M, Ciuffi A, Crisinel PA, Duppenthaler A, Dollenmaier G, Egger M, Elzi L, Fehr J, Fellay J, Francini K, Furrer H, Fux CA, Günthard HF (President of the SHCS), Haerry D (deputy of “Positive Council”), Hasse B, Hirsch HH, Hoffmann M, Hösli I, Huber M, Kahlert CR (Chairman of the Mother & Child Substudy), Kaiser L, Keiser O, Klimkait T, Kottanattu L, Kouyos RD, Kovari H, Ledergerber B, Martinetti G, Martinez de Tejada B, Marzolini C, Metzner KJ, Müller N, Nicca D, Paioni P, Pantaleo G, Perreau M, Polli Ch, Rauch A (Chairman of the Scientific Board), Rudin C, Scherrer AU (Head of Data Centre), Schmid P, Speck R, Stöckle M (Chairman of the Clinical and Laboratory Committee), Sultan-Beyer L, Tarr P, Thanh Lecompte M, Trkola A, Vernazza P, Wagner N, Wandeler G, Weber R, Yerly S.

*Funding:* the Swiss HIV Cohort Study is supported by the Swiss National Science Foundation (Grant #177499), and by the SHCS research foundation.

**Thailand:** Program for HIV Prevention & Treatment (PHPT).

*Participating hospitals:* Lamphun: Pornpun Wannarit; Phayao Provincial Hospital: Pornchai Techaku-nakorn; Chiangrai Prachanukroh: Rawiwan Hansudewechakul; Chiang Kham: Vanichaya Wanchai-tanawong; Phan: Sookchai Theansavettrakul; Mae Sai: Sirisak Nanta; Prapokklao: Chaiwat Ngampiyaskul; Banglamung: Siriluk Phanomcheong; Chonburi: Suchat Hongsiriwon; Rayong: Warit Karnchana-mayul; Bhuddasothorn Chacheongsao: Ratchanee Kwanchaipanich; Nakornping: Suparat Kanjana-vanit; Somdej Prapinklao: Nareerat Kamonpakorn, Maneeratn Nantarukchaikul; Bhumibol Adulyadej: Prapaisri Layangool, Jutarat Mekmullica; Pranangklao: Paiboon Lucksanapisitkul, Sudarat Watanayothin; Buddhachinaraj: Narong Lertpienthum; Hat Yai: Boonyarat Warachit; Regional Health Promotion Center 6, Khon Kaen: Sansanee Hanpinitsak; Nong Khai: Sathit Potchalongsin; Samutsakhon: Pimpraphai Thanasiri, Sawitree Krikajornkitti; Phaholpolphayuhasena: Pornsawan Attavinijtrakarn; Kalasin: Sakulrat Srirojana; Nakhonpathom: Suthunya Bunjongpak; Samutprakarn: Achara Puang-sombat; Mahasarakam: Sathaporn Na-Rajsima; Roi-et: Pornchai Ananpatharachai; Sanpatong: Noppadon Akarathum; Vachira Phuket: Weerasak Lawtongkum; Chiangdao: Prapawan Kheunjan, Thitiporn Suriyaboon, Airada Saipanya.

*Data management team:* Kanchana Than-in-at, Nirattiya Jaisieng, Rapeepan Suaysod, Sanuphong Chailoet, Naritsara Naratee, and Suttipong Kawilapat.

**Ukraine:** Pediatric HIV Cohort: Dr. T. Kaleeva, Dr. Y. Baryshnikova (Odessa Regional Centre for HIV/AIDS), Ruslan Malyuta (Perinatal Prevention of AIDS Initiative, Odessa), Dr. S. Soloha (Donetsk Regional Centre for HIV/AIDS), Dr. N. Bashkatova (Mariupol AIDS Center), Dr. I. Raus (Kiev City Centre for HIV/AIDS), Alla Volokha (Shupyk National Medical Academy of Postgraduate Education, Kiev), Dr. O. Glutshenko, Dr. Z. Ruban (Mykolaiv Regional Centre for HIV/AIDS), Dr. N. Prymak (Kryvyi Rih), Dr. G. Kiseleva (Simferopol), Dr. H. Bailey (UCL, London, United Kingdom).

Funding acknowledgement: PENTA Foundation.

**United Kingdom & Ireland:** Collaborative HIV Paediatric Study (CHIPS): CHIPS is funded by the NHS (London Specialised Commissioning Group) and has received additional support from Bristol-Myers Squibb, Boehringer Ingelheim, GlaxoSmithKline, Roche, Abbott, and Gilead Sciences. The MRC Clinical Trials Unit at UCL is supported by the Medical Research Council (https://www.mrc.ac.uk) programme number MC UU 12023/26. CHIPS Steering Committee: Hermione Lyall, Alasdair Bamford, Karina Butler, Katja Doerholt, Caroline Foster, Nigel Klein, Paddy McMaster, Katia Prime, Andrew Rior-dan, Fiona Shackley, Delane Shingadia, Sharon Storey, Gareth Tudor-Williams, Anna Turkova, Steve Welch. MRC Clinical Trials Unit: Intira Jeannie Collins, Claire Cook, Siobhan Crichton, Donna Dob-son, Keith Fairbrother, Diana M. Gibb, Lynda Harper, Ali Judd, Marthe Le Prevost, Nadine Van Looy. National Study of HIV in Pregnancy and Childhood, UCL: Helen Peters, Claire Thorne. *Participating hospitals:* Republic of Ireland: Our Lady's Children's Hospital Crumlin, Dublin: K Butler, A Walsh. United Kingdom: Birmingham Heartlands Hospital, Birmingham: L Thrasyvoulou, S Welch; Bristol Royal Hospital for Children, Bristol: J Bernatoniene, F Manyika; Calderdale Royal Hospital, Halifax: G Sharpe; Derby: B Subramaniam; Middlesex: K Sloper; Eastbourne District General Hospital, Eastbourne: K Fidler, Glasgow Royal Hospital for Sick Children, Glasgow: R Hague, V Price; Great Ormond St Hospital for Children, London: M Clapson, J Flynn, A Cardoso M Abou–Rayyah, N Klein, D Shingadia; Homerton University Hospital, London: D Gurtin, John Radcliffe Hospital, Oxford: S Yeadon, S Segal; King's College Hospital, London: C Ball, S Hawkins; Leeds General Infirmary, Leeds: M Dowie; Leicester Royal Infirmary, Leicester: S Bandi, E Percival; Luton and Dunstable Hospital, Luton: M Eisenhut; K Duncan, S Clough; Milton Keynes General Hospital, Milton Keynes: Dr. L Anguvaa, S Conway, Newcastle General, Newcastle: T Flood, A Pickering; North Manchester General, Manchester: P McMaster C Murphy; North Middlesex Hospital, London: J Daniels, Y Lees; Northampton General Hospital, Northampton: F Thompson; Northwick Park Hospital Middlesex; B Williams, S Pope; Nottingham QMC, Nottingham: L Cliffe, A Smyth, S Southall; Queen Alexandra Hospital, Portsmouth: A Freeman; Raigmore Hospital, Inverness: H Freeman; Royal Belfast Hospital for Sick Children, Belfast: S Christie; Royal Berkshire Hospital, Reading: A Gordon; Royal Children's Hospital, Aberdeen: D Rogahn L Clarke; Royal Edinburgh Hospital for Sick Children, Edinburgh: L Jones, B Offerman; Royal Free Hospital, London: M Greenberg; Royal Liverpool Children's Hospital, Liverpool: C Benson, A Riordan; Sheffield Children's Hospital, Sheffield: L Ibberson, F Shackley; Southampton General Hospital, Southampton: SN Faust, J Hancock; St George's Hospital, London: K Doerholt, K Prime, M Sharland, S Storey; St Mary's Hospital, London: H Lyall, C Monrose, P Seery, G Tudor-Williams; St Thomas' Hospital (Evelina Children's Hospital), London: E Menson, A Callaghan; Royal Stoke University Hospital, Stoke On Trent: A Bridgwood, P McMaster; University Hospital of Wales, Cardiff: J Evans, E Blake; Wexham Park, Slough: A Yannoulias.
